# Incorporating the human touch: piloting a curriculum for patient-centered electronic health record use

**DOI:** 10.1080/10872981.2017.1396171

**Published:** 2017-11-05

**Authors:** Wei Wei Lee, Maria L. Alkureishi, Kristen E. Wroblewski, Jeanne M. Farnan, Vineet M. Arora

**Affiliations:** ^a^ Department of Medicine, University of Chicago, Chicago, IL, USA; ^b^ Department of Pediatrics, University of Chicago, Chicago, IL, USA; ^c^ Department of Public Health Sciences, University of Chicago, Chicago, IL, USA

**Keywords:** Electronic health records, patient-centered care, communication skills, clinical skills training, curricular development

## Abstract

**Background**: Integrating electronic health records (EHRs) into clinical care can prevent physicians from focusing on patients. Despite rapid EHR adoption, few curricula teach communication skills and best practices for patient-centered EHR use.

**Objective**: We piloted a ‘Patient-centered EHR use’ curriculum, consisting of a lecture and group-observed structured clinical examination (GOSCE) for second-year students (MS2s).

**Design**: During the lecture, students watched a trigger tape video, engaged in a reflective observation exercise, and learned best practices. During the GOSCE, one of four MS2s interacted with a standardized patient (SP) while using the EHR. Third-year students (MS3s) received no formal training and served as a historical control group by completing the same OSCE individually. All students completed post-GOSCE/OSCE surveys. The SP evaluated GOSCE/OSCE performance.

**Results**: In 2013, 89 MS2s participated in the workshop and GOSCEs during their required Clinical Skills course and 96 MS3s participated in individual OSCEs during their end of year multi-station formative GOSCE exercise. Eighty MS2s (90%) and 88 MS3s (92%) post-GOSCE/OSCE surveys were analyzed. Compared to MS3s, significantly more MS2s rated their knowledge (19% vs 55%) and training (14% vs 39%) as good (≥4/5 point scale, *P *< .001 for both). Most learners (85% MS2s and 70% MS3s) thought training should be required for all students. SP ratings on GOSCE/OSCE performance was higher for the 20 MS2s compared to the 88 MS3 controls (73.5 [SD = 4.5] vs 58.1 [SD = 13.1] on 80 point scale, *P *< .001).

**Conclusions**: A short workshop and GOSCE were effective in teaching patient-centered EHR use. This curriculum is now a permanent part of our Clinical Skills course. Clerkship students who did not receive our curriculum may have been exposed to negative role-modeling on the wards. To address this, training residents and faculty on patient-centered EHR use skills should be considered.

**Abbreviations:** EHR: Electronic health record; EHR: Electronic health record; SP: Standardized patient

## Introduction

Integrating electronic health records (EHRs) into clinical care has altered patient-doctor communication [1–4]. Negative EHR-related behaviors, such as poor eye contact and prolonged screen gazing, can undermine patient-centered communication [2,5]. Conversely, when communication is patient-centered, patient outcomes, understanding, and adherence to treatment, and cost utilization can improve [6].

With increasing EHR adoption, physicians must balance clinical efficiency with maintaining meaningful interactions with patients [7–11]. In 2012, the Alliance for Clinical Education recommended medical schools develop clear sets of EHR-competencies and formally train students on these skills to prepare them for clinical practice [12]. The American Medical Association also recommends teaching physicians EHR-related communication skills, and recent editorials have called for curricula to teach these skills [13–16].

Despite these mandates, few trainees are formally taught how to use the EHR in a patient-centered way, and assessment methods are lacking. As a consequence, trainees rely on experiential learning and the hidden curriculum during clinical rotations to develop EHR-related communication skills, which may expose them to negative role modeling.

To address this gap, we piloted a patient-centered EHR use workshop and group-observed structured clinical examination (GOSCE) targeting second-year medical students (MS2s). Objectives were to: (1) highlight benefits of EHR use in clinical care; (2) identify barriers to patient-centered EHR communication; (3) introduce best practices; and (4) allow for skills practice and feedback.

Study aims were to: (1) compare GOSCE performance of MS2s, who received the curriculum, to individual OSCE performance of third years (MS3s), who received no formal training; and (2) compare MS2 and MS3 post-GOSCE/OSCE survey scores assessing knowledge, attitude, and skills.

Ethical approval was granted by the University of Chicago IRB.

## Methods

Investigators reviewed relevant literature to develop and implement the ‘Patient-centered EHR use’ lecture and GOSCE for MS2s at the Pritkzer School of Medicine in February 2013. Following the GOSCEs, written surveys and consent forms were distributed by non-faculty staff to all students. Participation had no impact on performance. Because all lectures are recorded and available online, lecture attendance is not required or tracked. However, we used an ‘intention to educate’ analysis assuming all MS2s received the lecture, either in person or remotely. This approach underestimates the true effect of actual participation in the curriculum. The University of Chicago Institutional Review Board approved this protocol.

### Curriculum design

**Figure 1.**
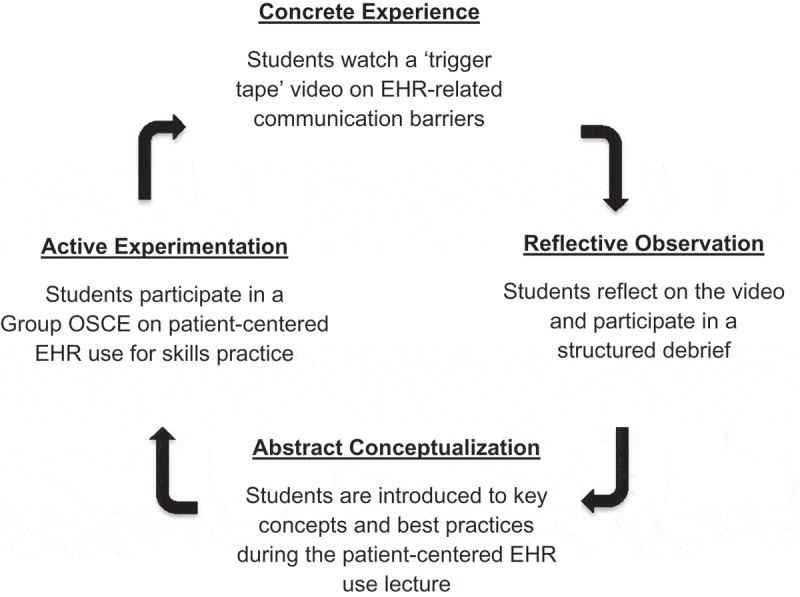
Applying Kolb’s experiential learning cycle to teach patient-centered electronic health record (EHR) use. We based our curriculum on Kolb’s experiential learning cycle.^17^ Second-year students (MS2s) participated in a ‘concrete experience’ by watching a ‘trigger tape’ video and engaged in ‘reflective observation’ during a structured video-debrief discussion. They attended a lecture introducing key concepts for ‘abstract conceptualization’ and participated in a Group Observed Structured Clinical Examination (GOSCE) for ‘active experimentation.’

The one-hour MS2 lecture was integrated into the Clinical Skills course. This was modeled on Kolb’s experiential learning theory to engage students in experiencing, reflecting, thinking, and acting to reinforce knowledge and skill acquisition [17] (Figure 1). Students participated in a ‘concrete experience’ by watching a trigger-video illustrating poor EHR communication skills [18]. They engaged in ‘reflective observation’ during a structured video-debrief discussion. The lecture provided ‘abstract conceptualization’ of patient-centered communication benefits and best practices. Lastly, students participated in the GOSCE for ‘active experimentation’ and feedback.

### Lecture

Students were taught the ‘HUMAN LEVEL’ mnemonic (Table 1) summarizing evidence-based best practices for patient-centered EHR use, such as ‘**U**sing the “triangle of trust”’ to position the screen where patient and provider can both see it. The lecture concluded with a second video depicting best practices [19]. Details of our full curriculum are published and accessible for reference [18,20].

**Table 1.** HUMAN^a^ LEVEL^b^ mnemonic: ten tips to enhance patient-centered electronic health record (EHR) use.

H	Honor the ‘Golden Minute’	Make the start of the visit completely technology free. Greet the patient, start with their concerns, and establish an agenda for the visit before engaging technology.
U	Use the ‘Triangle of Trust’	Create a triangle configuration that puts you, the patient, and the computer screen at each of the three corners. This allows you to look at both the patient and screen without shifting your body.
M	Maximize patient interaction	Encourage patient interaction with EHR. Pause for questions and clarification. Allow time for questions and to verify understanding.
A	Acquaint yourself with the chart	Review the chart before you enter the room to inform and contextualize your visit.
N	Nix the screen	When discussing sensitive information, completely disengage from the EHR (look at the patient, turn away from screen, take hands off keyboard, etc.)
L	Let the patient look on	Share things on the screen with your patients.
E	Eye contact	Maintain eye contact with patients as much as possible. Treat patient encounters as you would a conversation with friends or family members.
V	Value the computer	Praise the benefits of the EHR and take advantage of opportunities to use technology as a tool to engage patients (pull up lab result to review together, utilize graphics, etc.).
E	Explain what you’re doing	Be transparent about everything you do. Avoid long silences and aim for conversational EHR use by explaining what you are doing as you are doing it.
L	Log off	At the end of the visit, log off of the patient’s chart while they are still in the exam room. This assures patients that their medical information is secure.

^a^Developed by investigators.^b^Adapted from Kaiser Permanente^19^

### GOSCE/OSCE development

To provide students with a high-fidelity outpatient scenario, investigators worked with the University of Chicago’s EHR trainers to design a mock-chart in the training environment with five years of clinic notes and studies. During the GOSCE/OSCE, students logged into the mock chart, addressed the standardized patient’s (SP) chief concern, reviewed the EHR for relevant clinical information (i.e., labs, notes), and provided appropriate counseling. The SP received two hours of communication and evaluation training on how to use the SP evaluation tool, which is described below, to provide feedback on EHR utilization. All MS2s and MS3s were evaluated by the same SP, who was blinded to the student’s training status.

### MS2 group OSCE

Due to resource constraints in the preclinical curriculum, 89 MS2s participated in 15-minute 4–5 person group-OSCEs within one week of the lecture. One student interacted with the SP, remaining students observed, and a faculty member facilitated SP, peer and faculty feedback at the end of the case. The SP completed a written evaluation of the student’s performance using the SP evaluation tool.

### MS3 individual OSCE

In May 2013, 96 MS3s, who received no formal training on patient-centered EHR use, participated in the same OSCE as part of an end-of-year formative clinical OSCE experience. All MS3s participate in this multi-station OSCE exercise and receive feedback on their clinical skills with no formal grade assignment. All MS3s interacted with the SP individually and were given feedback using the SP evaluation tool.

### Evaluation of GOSCE/OSCE performance: SP evaluation tool

To assess student GOSCE/OSCE performance, we developed an SP evaluation tool based on the HUMAN LEVEL mnemonic highlighting best practices. Likert-style questions (15 questions, 1–5 scale; one question scored as no = 0 or yes = 5) assessed EHR-related communication skills with a score range of 15–80 points; higher scores indicated better performance.

### Surveys

All students completed paper-based post-GOSCE/OSCE surveys consisting of 20 Likert-style questions pertaining to knowledge, skill, confidence, and training on patient-centered EHR use. Likert responses at the high end (≥4 on a 5 point scale) were grouped to dichotomize data and report outcomes.

### Statistical analysis

Scores on the SP evaluation tool were summarized as means with standard deviations (SD), and the average scores compared between groups via two-sample *t* tests. Comparison of the score distribution between groups was assessed using Kolmogorov-Smirnov tests. Post-GOSCE/OSCE survey responses were tabulated and presented as frequency counts and percentages and compared between MS2 and MS3 groups using chi-square tests. Comparison of average scores between MS2s who directly interacted with the SP and MS2s who had no direct interaction was performed via two-sample *t* tests. Statistical analyses were conducted with Stata Version 13 (StataCorp, College Station, Texas).

## Results

All 89 MS2s and 96 MS3s participated in GOSCEs/OSCEs and all students completed post- GOSCE/OSCE surveys. 80 MS2s (80/89, 90%) and 88 MS3s (88/96, 92%) consented to participate in the study and were included in analysis. Of the 22 MS2s who directly interacted with the SP during the GOSCE, 20 (20/22, 91%) consented to the study and were included in analysis.

### GOSCE/OSCE outcomes

Twenty MS2s and 88 MS3s received SP evaluation scores (range 15–80). Overall, mean scores were significantly higher for MS2s than MS3s (73.5 [SD = 4.5] vs 58.1 [SD = 31.1], *P *< 0.001). MS2s scored higher than MS3s on 15/16 questions, including encouraging patient interaction with the EHR (4.7 [SD = 0.6] vs 2.9 [SD = 1.6], *P *< .001) and using the computer to promote communication and partnership (4.7 [SD = 0.5] vs 4.0[SD = 0.8], *P *< .001). MS2s performed better than MS3s on questions pertaining to computer positioning (4.7 [SD = 0.7] vs 3.1 [SD = 1.7]), eye contact (4.5 [SD = 0.7] vs 3.7 [SD = 0.8]), sharing the screen (4.7 [SD = 0.6] vs 2.9 [SD = 1.6]), and using the EHR to enhance patient-doctor communication (4.6 [SD = 0.6] vs 3.2 [SD = 1.2], *P *< .001 for all comparisons). In addition, beyond comparison of means, general differences in the distribution of scores between MS2s and MS3s were assessed using Kolmogorov-Smirnov tests and significant differences were consistently found. Results are summarized in Table 2.

**Table 2.** Standardized patient (SP) evaluation of student performance on Group and Individual Observed Structured Clinical Exam (GOSCE/OSCE).

	SP rating of student performance^a^			
SP evaluation tool question	MS2 (n = 20)	MS3 (n = 88)	P value^b^	P value^c^
Total score on 16 item SP evaluation tool	73.5 (4.5)	58.1 (13.1)	< .001	< .001
1. The student started the visit completely technology free, greeted me, and asked the reason of my visit.	4.9 (0.3)	3.7 (1.2)	< .001	< .001
2. The student positioned the computer screen between us like a triangle so I could see the screen and the student at the same time.	4.7 (0.7)	3.1 (1.7)	< .001	< .001
3. The student encouraged me to interact with the EHR during the visit, for example by showing me lab results on the screen, reviewing patient education materials, etc.	4.7 (0.6)	2.9 (1.6)	< .001	< .001
4. The student shared the screen with me and let me look on to see what he/she was doing on the EHR.	4.7 (0.6)	2.9 (1.6)	< .001	< .001
5. The student maintained eye contact during the visit.	4.5 (0.7)	3.7 (0.8)	< .001	< .001
6. When I discussed sensitive issues, the student completely focused on me instead of the EHR/computer (i.e., looked away from screen, took hands off the keyboard/mouse).	4.9 (0.4)	4.1 (0.8)	< .001	< .001
7. I can tell the student thinks the EHR is a positive thing, they value it, explained the benefits, and used it as a tool to engage me.	4.2 (0.6)	3.2 (1.0)	< .001	< .001
8. The student explained what they were doing on the computer/EHR as they were doing it.	4.5 (0.6)	3.4 (1.1)	< .001	.005
9. I felt the student promoted communication and partnership from me.	4.7 (0.5)	4.0 (0.8)	< .001	.001
10. I felt the student took time to listen to me as a person, I felt treated like an individual.	4.6 (0.5)	3.9 (0.8)	< .001	.01
11. I felt like the visit was an interactive and collaborative experience that was patient-centered, not provider or technology centered.	4.7 (0.5)	3.8 (0.8)	< .001	< .001
12. I felt the student took into account my beliefs, concerns, and personal circumstances.	4.7 (0.5)	4.0 (0.8)	< .001	.005
13. I felt treated with respect and dignity, like my voice was heard.	4.9 (0.3)	4.0 (0.7)	< .001	< .001
14. The student empowered me with information to make an educated individual treatment decision regarding my care.	4.7 (0.5)	3.9 (0.7)	< .001	< .001
15. How would you rate the student’s performance in using the EHR to enhance patient-provider communication during the standardized clinic visit?	4.6 (0.6)	3.2 (1.2)	< .001	< .001
16. The student closed my chart and logged out of the EHR when our visit was finished. [N (%) yes]	15 (75%)	76 (86%)	.21^d^	-

^a^Numbers in table are mean (SD) unless otherwise noted. The SP rated students using a 16-item evaluation tool. The SP rated student performance on a Likert scale of 1–5 points for each question, 1 being poor performance, 5 being exceptional performance. The maximum total score on the 16 questions was 80 points.

^b^From two-sample t test for comparison of means except as noted. Results were confirmed using the Wilcoxon rank-sum test (data not shown).

^c^From a two-sample Kolmogorov-Smirnov test for comparison of distributions.

^d^From a chi-square test

### Post-GOSCE/OSCE survey

After the workshop and GOSCE/OSCE, significantly more MS2s (44/80, 55%) rated their knowledge as good compared to untrained MS3s (17/88, 19%) (*P *< .001). Thirty-one (31/80, 39%) MS2s rated their training as good, compared with 12 MS3s (12/88, 14%) (*P *< .001). While 26% (21/80) of MS2s reported they were confident compared to 16% (14/88) of MS3s, this was not statistically significant (*P *= .10). Few MS2s or MS3s rated their skill level in using the EHR in a patient-centered way as competent (10% [8/80] vs 9% [8/88] respectively, *P *= .84). Most MS2s and MS3s agreed that the topic was important to their training (71% [57/80] and 56% [49/88] respectively, *P *= .04]. Overall, 85% (68/80) of MS2s and 70% (62/88) of MS3s agreed training should be required for all students (Table 3).

**Table 3.** Post-observed structured clinical exam (OSCE) survey results: comparison of MS2 and MS3 students’ knowledge, attitudes, and skills pertaining to ‘patient-centered electronic health record (EHR) use’^a^.

		% (n) of students^b^		
Post-OSCE survey questions	Responses of interest	MS2 (n = 80)	MS3 (n = 88)	P value^c^
1. How would you rate your knowledge regarding patient-centered EHR use?	Good (≥4 on 5 point scale)	55% (44)	19% (17)	< .001
2. How would you rate your skill level in integrating the EHR in a patient-centered manner?	Competent (≥4 on 5 point scale)	10% (8)	9% (8)	.84
3. How confident are you using the EHR in a patient-centered manner?	Moderately (≥4 on 5 point scale)	26% (21)	16% (14)	.10
4. How would you rate your training on using the EHR in a patient-centered manner?	Good (≥4 on 5 point scale)	39% (31)	14% (12)	< .001
5. Training on patient-centered EHR use should be required for all medical students.	Agree (≥4 on 5 point scale)	85% (68)	70% (62)	.02

^a^80/89 of MS2s and 88/96 MS3s post-OSCE surveys were analyzed. The post-OSCE survey consisted of 20 questions. We highlighted representative questions and responses.

^b^Percent of students with one of the responses of interest.

^c^From a chi-square test. Results confirmed using Fisher’s exact test (data not shown).

Survey results of MS2s who directly interacted with the SP during the GOSCE (n = 20) showed no significant difference in self-reported skill (2.4 [SD = 1.0] vs 2.4 [SD = .8], P = 0.95), confidence (3.3 [SD = .7] vs 3.1 [SD = .7], *P *= 0.39), and training (3.4 [SD = .7] vs 3.1 [SD = 1.0], *P *= 0.27) when compared to peer student observers (n = 60). However, the two groups differed when asked about knowledge; MS2s interacting with the SP rated higher knowledge compared to observers (3.9 [SD = .7] vs 3.5 [SD = .9], *P *= .04).

## Discussion

To our knowledge, this is the first study to show that a formal lecture and GOSCE are effective in teaching medical students how to use the EHR to promote patient-centered communication. We found that MS2s who received a brief one-hour lecture performed better on a GOSCE than more clinically experienced MS3s who received no formal training. We chose to deliver the curriculum to MS2s to allow them to incorporate GOSCE feedback to improve communication with real patients during their clerkships. Due to the success of the pilot, our curriculum has been formally incorporated into the MS2 Clinical Skills Course. Importantly, our findings suggest that the current method of relying on experiential learning during clerkships is not sufficient to master these patient-centered EHR use skills.

It is worth considering why MS2 confidence and self-reported skill levels were not higher after the curriculum, despite higher ratings by SPs. First, MS2s are still novices at interacting with patients, and this exercise may be inherently complex as it requires effective use of the EHR while integrating patient counseling. Students may require repeated interactions and re-evaluation to achieve a higher level of confidence. It is also possible that MS2s forgot the skills after the workshop or did not come to the lecture. Historically, approximately half the class watches recordings of lectures in lieu of attending class, but this is not tracked due to privacy reasons. Overall, our lecture attendance rate is consistent with those reported in the literature [21]. While we conducted an ‘intention to educate’ analysis assuming all students received the curriculum, it is possible that some students did not watch the recorded lecture or that watching a video was less effective than in-person participation. Further studies should investigate if these learning strategies impact GOSCE performance.

Our curriculum has potential uses outside of medical student training. Currently, most physicians are required to attend mandatory EHR training sessions that focus on EHR functionality but do not address patient-doctor communication. Given the focus on patient experience in healthcare, incorporating best practices of patient-centered EHR use into mandatory EHR training can improve the quality of patient-doctor communication. Presently, we have expanded our patient-centered her-use curriculum to internal medicine and pediatrics residents and faculty; validation of our evaluation tools is underway. Lastly, we are exploring the use of direct observation in real clinic interactions to assess MS3, resident, and faculty’s patient-centered EHR use.

Our study has limitations that open avenues for further research. MS2s participated in GOSCEs, which is common practice in undergraduate medical education due to limited time and financial resources. Future research should look at MS2 individual OSCE performance, as our study suggests active OSCE participation may promote higher rates of knowledge acquisition, perhaps due to the individual feedback obtained. Another limitation is the self-reported nature of responses. While student survey responses were in line with SP evaluations, validating SP evaluations and parallel evaluations of student GOSCE performance by faculty observers is needed. Future studies can also evaluate the impact of direct participation in the live-lecture versus watching the recording online. In spite of these limitations, our findings provide a real-world estimate of the effectiveness of this curriculum, which is valuable to other medical schools that do not require lecture attendance. Moreover, since we used an ‘intention to educate’ analysis, assuming all students received the lecture; our findings may under-estimate the true effects of receiving a formal curriculum.

In summary, we successfully implemented a new curriculum and GOSCE that led to increased knowledge, skills, and performance in patient-centered EHR communication. This innovation can be expanded to teach residents and faculty how to remain focused on the patient while managing the demands of the EHR.
